# Supplementary material for: What is a minimal clinically important difference for clinical trials in patients with disorders of consciousness? a novel probabilistic approach
Source: PLoS One. 2023 Aug 24;18(8):e0290290. doi: 10.1371/journal.pone.0290290 (PMC10449161; doi:10.1371/journal.pone.0290290)
Supplement: Attachment — Submitted filename: Reviewer_feedback_v2.docx [file pone.0290290.s001.docx]

**Reviewer 1:**

**Monti et al. present a paper in which they develop a probabilistic score of the minimal clinically important difference (MCID) after a therapeutic intervention in patients with disorders of consciousness (DoC). They elegantly introduce the intrinsic challenges of identifying this threshold (i.e., DoC patients are either unaware or low-responsive, which means they are unable to provide subjective reports of perceived benefit), and they continue to solve this problem in a most convincing way by benchmarking their probabilistic MCID score against the clinical gold standard scale of DoC, the Consciousness Recovery Scale revised (CRS-R).**

**The paper is timely, important, and well-written. I am convinced that it will become an invaluable and well-cited resource for therapeutic DoC trials in the future.**

We appreciate the positive feedback.

**I have only some very minor suggestions:**

**1. According to the CRS-R, visual fixation is enough to diagnose MCS- but other sources have argued that visual fixation might be a reflexive behavior and that visual pursuit is required for an MCS- diagnosis. What is the authors’ opinion?**

We agree entirely with the reviewer on the substance of the issue that visual fixation is, by some clinicians and investigators, consistent with a VS/UWS diagnosis. There are several other behaviors on the CRS-R (e.g., localization to auditory stimuli and consistent command following) whose related diagnoses, VS/UWS and MCS+, respectively, have been debated. In a field like DoC, where subtle behaviors can have a profound effect on clinical care, diagnostic criteira are likely to continue to change. One of the strengths of our method is its flexibility which allows for instant re-characterization of behaviors and generation of new probability tables.

For instance, changing one single digit in the code allows creating a new Table 3 where Visual Fixation is no longer a threshold behavior for MCS-. As can be seen below (“new Table 3” on the left, “original Table 3” on the right), it does change the pMCID for VS and MCS- (albeit not much).

| **Table 3 if visual fixation is VS** | | | | |  | | **Original Table 3 (as in the manuscript)** | | | | |
| --- | --- | --- | --- | --- | --- | --- | --- | --- | --- | --- | --- |
|  | Observed change in CRS-R total score | | |  | |  | | Observed change in CRS-R total score | | |  |
| Diagnosis | 1 point | 2 points | 3 points |  | | Diagnosis | | 1 point | 2 points | 3 points |  |
| VS | 69.51 | 79.11 | 86.79 |  | | VS | | 72.78 | 82.36 | 89.56 |  |
| MCS- | 76.45 | 81.95 | 86.84 |  | | MCS- | | 74.86 | 80.43 | 85.47 |  |
| MCS+ | 32.73 | 39.48 | 46.34 |  | | MCS+ | | 32.73 | 39.48 | 46.34 |  |
| *Average* | *59.56* | *66.84* | *73.32* |  | | *Average* | | *60.12* | *67.42* | *73.79* |  |

To the substance of the reviewer’s question, however, in the present manuscript we adhere to published diagnostic criteria for MCS (e.g., Giacino et al. 2002) which are supported by the American Academy of Neurology/American Congress of Rehabilitation Medicine/National Institute for Disability, Independent Living, and Rehabilitation Research DoC clinical guidelines (Giacino et al., 2018) as well as the European Academy of Neurology (Kondziella, et al 2020) and the UK Royal College of Physicians (2020) clinical guidelines consider Visual Fixation as a threshold behavior for MCS-.

We have, however, added an in-text note that brings up the point as suggested by the reviewer (p.14):

“*While it has now been over 20 years from the initial distinction of MCS from VS [28], the nosology of DOC has been refined over time [34] and the relationship between individual items on the CRS-R scale (e.g., visual fixation, localization to noxious stimulation, consistent response to command) and diagnosis is still being discussed (cf., [46, 47]) and could well change as more evidence and novel techniques are introduced*.”

**2. Being a reviewer from Europe, I am used to the term “VS/UWS” instead of “VS” given the latter’s negative connotations. “VS” is the established term in the US, but for the sake of international outreach, the authors might want to consider changing the terminology. (I know, I know… “VS/UWS” sounds awkward, but still.)**

Thank you for the suggestion. We have now added the following when we introduce the DOC definitions (p. 5-6; addition in bold):

“*when a patient considered to be in a Vegetative State (VS****; also referred to as Unresponsive Wakefulness Syndrome; UWS [33]****)*”

**3. The differentiation between VS/UWS and MCS is most probably gradual (continuous) rather than binary (all-or none), and the level of arousal and responsiveness in DoC patients can be subject to fluctuations over very short time spans (minutes, hours), which the MCID score for obvious reasons cannot take into account. Perhaps the authors might want to include a statement about this limitation.**

We agree, given that our pMCID is based on “all-or-none” categories it does fail to capture within-category gradation which are likely to exist. We note, however, this is a limitation in the “input structure” (i.e., the fact that the threshold-behaviors are based on big categories) not necessarily in our method. In fact, the ability to incorporate advanced in the field is a strength of our approach. To reflect this feedback, we have added the following at the end of the discussion section (p.16):

“*Finally, by virtue of calculating the pMCID on the basis of relatively large “all-or-none” diagnostic categories, this approach fails to capture finer changes in level of consciousness and what is likely to be a more continuous spectrum of impairments [57]. However, we highlight that this limitation is due to the choice of using well-established diagnostic markers as the basis for threshold behaviors. As the definition of what should count as a change that “would mandate […] a change in the patient's management” [5] becomes more sophisticated, the pMCID method can be adapted to reflect such finer gradations.*”

**4. Conclusion, line 307: “This is the first study reporting…” – I am not sure I would call this a “study” since the authors did not collect original data or tested their score in a patient cohort. I would suggest changing the sentence to “We report…” or “This is the first paper reporting…”**

We edited it to read “*This is the first* ***paper***…”

**5. Lines 129/1300: Small typo, should be ”In what follows, we..”**

Corrected!

**6. The authors provide a link to OSF with their code to calculate the MCID score, which is excellent. A graphical depiction of the idea/concept/design of the MCID score and its calculation would be great. It's optional, but the visual appeal of the paper could be increased a lot with a nice illustration.**

Thank you for the suggestion. We generated Figure 1, shown here, to give a depiction of how the pMCID is calculated.

***Figure 1.*** *Depiction of a sample calculation of the average probabilistic minimal clinically important difference (pMCID) for a 1-point change in CRS-R total score across all diagnoses. In brief, the pMCID for a 1-point change is calculated by taking the ratio of the number of 1-point changes that result in a new threshold behavior (numerator) over the number of all possible 1-point changes (denominator). (Note: while not depicted, the calculation also includes “net” 1-point changes; e.g., where a 1-point decrease is observed in one subscale and a 2-point increase is observed on a different subscale; see methods for full detail.)*

**Reviewer 2:**

**I read Dr. Monti et al manuscript with interest. This is an extremely important topic, and I am grateful for the authors addressing and proposing their approach of pMCID in DOC patients. I congratulate them on this very well written paper.**

**The two challenges as explained elegantly by the authors in the MCID concept in DOC patients are: 1/ the subjective perception of improvement by the patient is absent or not accessible, 2/ the CRS-R is not designed to be proportional and/or consistent. Thus, the authors have developed a probabilistic approach to establishing MCID (pMCID) for the CRS-R. The authors selected 8 threshold behaviors that reflect the behavioral diagnostic of each DOC (VS, MCS-, and MCS+). Tables 3 &4 reflect the probability of patient attaining a new threshold behavior given an observed 1,2, and 3-point change in VS, MCS-, and MCS+. I do favor including consistent movement to command to index eMCS and I appreciate the authors adding the probabilistic model in table 4. The authors have also made the code publicly available to calculate pMCID to reproduce the results. This proposed approach doesn't overestimate the clinically meaningful change.**

We sincerely appreciate the positive feedback on the contents and on the writing.

**I have the following minor suggestions/comments:**

**#Minor: Shouldn't MCID refer to a minimal clinically important difference? and not significant difference (in some parts of the manuscript).**

Thank you, we have standardized our text to always read “important” which is indeed the appropriate term.

**#The changes in CRS-R, especially in the acute phase after brain injury, may in part be related to the changes of sedation, intracranial pressure, hemodynamics, and seizures among others. I would encourage the authors to address this point specifically. I understand that the CRS-R is not as validated to be used in the acute phase as it is for the subacute and chronic phases after injury. So perhaps the use of pMCID should be limited to those without the confounders mentioned above.**

This is a good suggestion. Indeed, the pMCID, in this respect, is tethered to “valid application” of the CRS-R assessment. We added new text to highlight this important limitation to the valid application of pMCID (in the discussion, p.15-16):

“*It should also be considered that, as conceived presently, our approach is valid only inasmuch as the CRS-R is applied appropriately. Specifically, the pMCID has no embedded mechanism to account for confounding factors such as those typical of the acute care setting (e.g., sedation, intracranial pressure, hemodynamics, etc). In this respect, our approach relies on being able to abide by well-recognized best practices for CRS-R assessments [12], which are difficult to meet in acute care, potentially limiting the appropriateness of the pMCID to clinical trials in the subacute and chronic phases of the disease. In partial mitigation of this concern, the CRS-R now contains completion codes aimed at identifying (and removing) potential confound sources [54]. In addition, our approach should also be easy to translate to shortened protocols directly derived from the CRS-R, such as the CRSR-FAST [55] and the SECONDs [56].*”

**#The approach in its current version does not address the clinically meaningful change to recovered patients (although might introduce some biases), their families, clinicians, and others. The authors have addressed this point in their discussion.**

We agree entirely, the present manuscript uses, as a spring board, the well-established diagnostic criteria, but we hope to refine this in the future by incorporating additional stakeholders who might help define what should count as a “important change”. We added a sentence, in the discussion, to highlight this (underappreciated) point (p. 14):

“*Furthermore, while we currently focus exclusively on diagnostic behaviors as markers of clinically important changes, our method could be easily extended to include the views of other stakeholders, such as family members, on which items they would consider particularly valuable.*”

**#Additionally, the current approach doesn't take into account the covert responses observed with neuroimaging or electrophysiology (CMD), or covert tracking using the video eye-tracking devices (PMID: 37076304) - I also suggest adding the latter since the eye-tracking response is one of the most important elements of CRS-R. Future studies should incorporate the covert responses to build up on the proposed framework.**

Thank you for bringing this new work to our attention, it fits very well in our discussion of non-standard approaches to detecting responsiveness in DOC and we have now incorporated it in the discussion section (p.15) as follows:

“***In addition****, we also note that while the pMCID developed above relies on the observation of motor-response-dependent (responsive) behaviors.* ***Yet,*** *a* ***growing*** *literature demonstrates that it is possible for DOC patients to be unresponsive in traditional bedside assessments while demonstrating responsiveness through neuroimaging and electrophysiology-based approaches [50]* ***as well as enhanced technology-based monitoring (e.g., video eye-tracking) [51]****. In as much as it is possible to map* ***such*** *covert responses to items of the CRS-R (e.g.,* ***eye-tracking,*** *response to command), the approach developed above should generalize to patients with covert awareness or cognitive-motor dissociation [52].*”

**#I would suggest adding a paragraph on the discussion on how the authors visualize the use of this tool in future clinical studies and trials, and perhaps give an example. This will help visualize the importance of this tool.**

This is a good suggestion, also because there might be multiple uses for this tool. We have added a brief example in the conclusion of the paper, as follows (p. 16):

“*As per Table 3, for example, a 2-point change in total CRS-R score is 82% likely to be associated with a clinically important change in VS patients. Thus, if an intervention gave rise to a 2-point change across a sample of patients, it would have to be considered successful. In addition, the tool could also be modified to allow research teams to create pMCIDs tailored to their research question (i.e., by specifying threshold behaviors that are relevant to their specific research question).*”
